# A case-control pilot study of x-ray measurements between the affected and unaffected foot in patients with unilateral hallux limitus

**DOI:** 10.1186/1757-1146-8-S2-P6

**Published:** 2015-09-22

**Authors:** Andrew F Knox, Alan R Bryant

**Affiliations:** 1 Podiatric Medicine Unit, School of Surgery, The University of Western Australia, Crawley, WA, 6009, Australia

**Keywords:** hallux limitus, hallux rigidus, aetiology, radiographic analysis

## Background

Controversy exists regarding the structural and functional causes of hallux limitus including metatarsus primus elevatus, long first metatarsal, first ray hypermobility, shape of the first metatarsal head and the presence of hallux interphalangeus. Papers have reported on the radiographic evaluation of these measurements in feet affected by hallux limitus, however no study to date has made direct comparisons between the affected and unaffected foot in a group of patients with unilateral hallux limitus. This case-control pilot study aimed to establish if any such differences existed.

## Methods

Dorsoplantar and lateral weightbearing x-rays of both feet in 30 patients with unilateral hallux limitus were assessed for grade of disease, lateral intermetatarsal angle, metatarsal protrusion distance, plantar gapping at the first metatarso-cuneiform joint, metatarsal head shape and hallux abductus interphalangeus angle. Data analysis was performed using SPSS version 22.0.0.0 (SPSS Science, Chicago, IL).

## Results

Mean radiographic measurements for both affected and unaffected feet demonstrated that metatarsus primus elevatus, a short first metatarsal, first ray hypermobility, a flat metatarsal head shape and hallux interphalangeus were prevalent in both feet. There was no statistically significant difference found between feet for any of the radiographic parameters measured (Mann-Whitney U Tests, Independent Samples T-Tests and Pearson Chi Square Test p > 0.05).

## Conclusion and clinical relevance

No significant differences exist in the presence of structural risk factors examined between the affected and unaffected foot in patients with unilateral hallux limitus. The influence of other intrinsic factors including footedness and family history should be investigated further.

